# GWEP Partnerships With Community-Based Organizations to Advance Age-Friendly Health Systems

**DOI:** 10.1093/geroni/igaa057.1774

**Published:** 2020-12-16

**Authors:** Leland Waters, Nina Tumosa

## Abstract

From the onset of the Health Resources and Services Administration’s Geriatrics Workforce Enhancement Program (GWEP), project initiatives have been developed to collaborate with Area Agencies on Aging and other community-based organizations (CBOs) serving the older adult community to improve health outcomes. The current GWEP funding cycle includes an initiative to further the principles of Age-Friendly Health Systems. Having built relationships within the aging services community, GWEPs are well positioned to deliver educational interventions framed around the 4M pillars of age-friendly healthcare. Three GWEPs are working with CBOs to implement and test interventions that address the interactive 4Ms. One GWEP has created an Age Friendly Community Education series in conjunction with their CBO partners in a low-income urban setting. Another GWEP has secured age friendly designation for its teams and worked with a coalition of CBOs to obtain both age and dementia-friendly designations for its home city of Springfield, MA. A third GWEP is implementing age-friendly practice workflows in programs that connect primary care and community-based organizations to address specific needs of an underserved older population. By integrating CBOs within the age-friendly health system framework, we can better create sustainable partnerships with health systems to improve health outcomes for our elders.

